# Global and Regional Estimates for Subtype-Specific Therapeutic and Prophylactic HIV-1 Vaccines: A Modeling Study

**DOI:** 10.3389/fmicb.2021.690647

**Published:** 2021-07-15

**Authors:** Ramyiadarsini Elangovan, Michael Jenks, Jason Yun, Leslie Dickson-Tetteh, Shona Kirtley, Joris Hemelaar, WHO-UNAIDS Network for HIV Isolation and Characterisation, Alash’le G Abimiku

**Affiliations:** Institute of Human Virology, University of Maryland, Baltimore, MD, USA (A G Abimiku, J Carr); Gede Foundation, Abuja, Nigeria (S Agwale); Public Health Agency of Canada, Ottawa, ON, Canada (C Archibald, J Brooks, M Ofner, P Sandstrom, J Stokes); Tel-Aviv Sourasky Medical Center, Tel-Aviv, Israel (B Avidor); Ministerio de Salud, Córdoba, Argentina (M G Barbás); Institut Pasteur, Paris, France (F Barre-Sinoussi, E Menu); Ministry of Health, Entebbe, Uganda (B Barugahare); National Reference Laboratory on HIV/AIDS, Institut Pasteur d’Algérie, Algiers, Algeria (E Belabbes); World Health Organization, Geneva, Switzerland (S Bertagnolio); Office of the Global AIDS Coordinator, Washington, DC, USA (D Birx); The D I Ivanovsky Institute of Virology, Moscow, Russia(A F Bobkov); Noguchi Memorial Institute for Medical Research, University of Ghana, Accra, Ghana (J Brandful); University of Cape Town, Cape Town, South Africa (H Bredell, C Rademeyer, P Selhorst, D Stewart, C Williamson); Abbott Laboratories, Chicago, IL, USA (C A Brennan); National Institute of Public Health, Prague, Czech Republic (M Bruckova, M Linka); AIDS Reference Center, National Cancer Institute “Fond. G. Pascale”, Naples, Italy (L Buonaguro, F Buonaguro); National AIDS Center, Istituto Superiore di Sanità, Rome, Italy (S Buttò, B Ensoli); Institute of Tropical Medicine, Antwerp, Belgium (A Buvé, G van der Groen); University of Washington School of Medicine, Seattle, WA, USA (M Campbell, C Celum, J Herbeck, E Kahle, J Lingappa, J Mullins, M Rolland, C Rousseau); St Vincent’s Hospital, Sydney, Australia (A Carrera, P Cunningham); University of Buenos Aires, Buenos Aires, Argentina (M Carrillo, H Salomon); Harvard T H Chan School of Public Health, Boston, MA, USA (B Chaplin, P Kanki); Gheskio Center, Port-au-Prince, Haiti (M Charles, C Nolte, J Pape); Aristotle University of Thessaloniki, Thessaloniki, Greece (D Chatzidimitriou, A Papa); Chinese Academy of Medical Sciences, Peking Union Medical School, Beijing, China (Z Chen, L Zhang, R Zhang); Fukuoka Institute of Health and Environmental Sciences, Kyushu University Hospital, Dazaifu, Japan (K Chijiwa); The Kirby Institute, Sydney, NSW, Australia (D Cooper, T Kelleher, A Pinto, A Shaik); Faculté des Sciences de la Santé, Université de Lomé, Lomé, Togo (A Dagnra); University College Dublin, Dublin, Ireland (C de Gascun); Instituto de Salud Carlos III, Madrid, Spain (J Del Amo, E Delgado, R Najera, L Pérez-Álvarez, M Thomson); Chemotherapeutisches Forschungsinstitut, Georg-Speyer-Haus, Frankfurt, Germany (U Dietrich); Pathology West, Westmead Hospital, Westmead, NSW, Australia (D Dwyer, K Thapa, T Tran); Centers for Disease Control and Prevention, Atlanta, GA, USA (D Ellenberger, P N Fonjungo, M A Rayfield, K E Robbins, S Subbarao, C Yang); Harvard School of Public Health, Boston, MA, USA (M Essex, V Novitsky, A D Sarr); Duke University Medical Center, Durham, NC, USA (F Gao); University of Bordeaux, Bordeaux, France (H Fleury); Montpellier University Hospital, Montpellier, France (V Foulongne, P van de Perre); National AIDS Research Institute, Pune, India (D A Gadkari); Complejo Hospitalario Universitario de Granada, Granada, Spain (F García); Royal Prince Alfred Hospital, Sydney, Australia (R Garsia, H Salem); HIV Laboratory Programme on AIDS/AFRO, World Health Organisation, Ouagadougou, Burkina Faso (G M Gershy-Damet); London School of Hygiene and Tropical Medicine, London, UK (J R Glynn); University College London, London, UK (R Goodall); National HIV Reference Laboratory, Ministry of Health, Tel Aviv, Israel (Z Grossman, O Mor); Instituto Oswaldo Cruz, FIOCRUZ, Rio de Janeiro, Brazil (M L Guimarães, M Morgado); University of Pennsylvania, Philadelphia, PN, USA (B Hahn); Amsterdam Institute for Global Health and Development, Amsterdam, Netherlands (R L Hamers, T Rinke de Wit); Robert Koch Institute, Berlin, Germany (O Hamouda, C Kucherer); Yamanashi Medical University, Yamanashi, Japan (R Handema, M Ito); National Center for AIDS/STD Control and Prevention, China CDC, Beijing, China (X He, Y Shao, J Xu); Aaron Diamond AIDS Research Center, The Rockefeller University, New York, NY, USA (D D Ho, L G Kostrikis); Ramón y Cajal Research Institute, Hospital Universitario Ramón y Cajal de Madrid, Madrid, Spain (A Holguin); University of North Carolina, Chapel Hill, NC, USA (M Hosseinipour); National Institute for Communicable Diseases, Johannesburg, South Africa (G Hunt); Charles Nicolle Hospital, Tunis, Tunisia (M Kacem, A Moussi, M Nasr, A Slim); Medical Research Council, Entebbe, Uganda (P Kaleebu, C Parry); Vanderbilt Institute for Global Health, Vanderbilt University School of Medicine, Nashville, TN, USA (M Kalish); University of Malaya, Kuala Lumpur, Malaysia (A Kamarulzaman, K-K Tee); Institute for Molecular Biology and Genetics and Medical College, Seoul National University, Seoul, Korea (C Kang); Gamaleya Center for Epidemiology and Microbiology, Moscow, Russian (E Karamov); National Reference Laboratory, Kigali, Rwanda (J-C Karasi); Emory University School of Medicine, Atlanta, GA, USA (K Kayitenkore); HIV/AIDS Collaboration, Nonthaburi, Thailand (D Kitayaporn); Karolinska Institute, Huddinge University Hospital, Stockholm, Sweden (C Lara); Los Alamos National Laboratory, Los Alamos, NM, USA (T Leitner); National Institute for Health and Wellfare, Helsinki, Finland (K Liitsola, M Salminen); National Autonomous University of Honduras, Tegucigalpa, Honduras (I Lorenzana de Rivera); Academic Medical Center, University of Amsterdam, Amsterdam, Netherlands (V Lukashov); Hadassah U Hospital, Jerusalem, Israel (S Maayan); New York University School of Medicine, New York, NY, USA (L Mayr, P Nyambi); Henry M Jackson Foundation for the Advancement of Military Medicine, Bethesda, MD, USA (F McCutchan); Centre Muraz, Bobo-Dioulasso, Burkina Faso (N Meda); Muhimbili University of Health Sciences, Dar-es-salaam, Tanzania (F Mhalu, D Mloka, F Mosha, W Urassa); University of Edinburgh, Edinburgh, UK (J L Mokili); Montpellier University Hospital, Montpellier, France (B Montes, M Segondy); Institute of Human Virology, Abuja, Nigeria (N Ndembi); University of Washington, Seattle, WA, USA (J R Neilson); University of Hawaii, Honolulu, HI, USA (V R Nerurkar); University Clinic Heidelberg, Heidelberg, Germany & Lighthouse Trust, Lilongwe, Malawi (F Neuhann); Research Institute for Tropical Medicine, Muntinlupa City, Manila, Philippines (F J Paladin, M L Santiago); Data First Consulting, Inc, Belmont, CA, USA (N Parkin); University of Montpellier, Montpellier, France (M Peeters, N Vidal); Centre de Recherche Public-Santé, Luxembourg, Luxembourg (A Pelletier, J Servais); Africa Health Research Institute, Durban, KwaZulu-Natal, South Africa & Division of Infection and Immunity, University College London, London, UK (D Pillay); Institute of International Health, University of Tokyo, Tokyo, Japan (T D Quang); University of Sydney, Sydney, NSW, Australia (F Raikanikoda); Institut Pasteur du Cambodge, Phnom Penh, Cambodia (J-M Reynes); University of Alabama at Birmingham, Birmingham, AL, USA (J Salazar-Gonzales); Auckland City Hospital, Auckland, New Zealand (B Schroeder); Uganda Virus Research Institute, Entebbe, Uganda (S Sempala); Instituto Nacional de Câncer, Rio de Janeiro, Brazil (M A Soares); Kenya Medical Research Institute, Nairobi, Kenya (E Songok); National HIV Repository and Bioinformatic Center, Siriraj Hospital, Mahidol University, Thailand (R Sutthent); Laboratory of Viral Pathogenesis, Kyoto University, Kyoto, Japan (J Takehisa); Federal University of Rio de Janeiro, Rio de Janeiro, Brazil (A Tanuri); Aino Health Science Center and Aino University, Tokyo, Japan (H Ushijima); Rega Institute for Medical Research, KU Leuven, Belgium (K van Laethem, E van Wijngaerden, A-M Vandamme, J Vercauteren); Department of Medicine, Blantyre, Malawi (J van Oosterhout); Stichting HIV Monitoring, Amsterdam, Netherlands (A van Sighem); Health Protection Scotland, Glasgow, UK (L Wallace); and Ethiopian Health & Nutrition Research Institute, Addis Ababa, Ethiopia (D Wolday).; ^1^Nuffield Department of Population Health, University of Oxford, Oxford, United Kingdom; ^2^Nuffield Department of Orthopaedics, Rheumatology and Musculoskeletal Sciences, Centre for Statistics in Medicine, Botnar Research Centre, University of Oxford, Oxford, United Kingdom

**Keywords:** HIV, subtype, recombinant, CRF, URF, vaccine

## Abstract

Global HIV-1 genetic diversity forms a major obstacle to the development of an HIV vaccine. It may be necessary to employ subtype-specific HIV-1 vaccines in individual countries according to their HIV-1 subtype distribution. We estimated the global and regional need for subtype-specific HIV-1 vaccines. We took into account the proportions of different HIV-1 variants circulating in each country, the genetic composition of HIV-1 recombinants, and the different genome segments (*gag*, *pol*, *env*) that may be incorporated into vaccines. We modeled different scenarios according to whether countries would employ subtype-specific HIV-1 vaccines against (1) the most common subtype; (2) subtypes contributing more than 5% of HIV infections; or (3) all circulating subtypes. For therapeutic vaccines targeting the most common HIV-1 subtype in each country, 16.5 million doses of subtype C vaccine were estimated globally, followed by subtypes A (14.3 million) and B (4.2 million). A vaccine based on *env* required 2.6 million subtype E doses, and a vaccine based on *pol* required 4.8 million subtype G doses. For prophylactic vaccines targeting the most common HIV-1 subtype in each country, 1.9 billion doses of subtype A vaccine were estimated globally, followed by subtype C (1.1 billion) and subtype B (1.0 billion). A vaccine based on *env* required 1.2 billion subtype E doses, and a vaccine based on *pol* required 0.3 billion subtype G doses. If subtype-specific HIV-1 vaccines are also directed against less common subtypes in each country, vaccines targeting subtypes D, F, H, and K are also needed and would require up to five times more vaccine doses in total. We conclude that to provide global coverage, subtype-specific HIV-1 vaccines need to be directed against subtypes A, B, and C. Vaccines targeting *env* also need to include subtype E and those targeting *pol* need to include subtype G.

## Introduction

Thirty-eight million people globally were living with HIV in 2019 (UNAIDS, 2020). Despite the increased availability of antiretroviral therapy, there were 690,000 deaths and 1.7 million new infections in 2019 (UNAIDS, 2020). A globally effective preventative HIV vaccine is likely necessary to end the HIV pandemic (Fauci, 2017). Furthermore, a therapeutic vaccine that augments the immune system of HIV-infected individuals may reduce the need for antiretroviral therapy (Dorrell, 2005). However, a key stumbling block to the development of an HIV vaccine is the extensive global genetic diversity of HIV (Barouch, 2008; Hemelaar, 2012, 2013).

HIV has its origins in the zoonotic transmission of Simian Immunodeficiency Virus (SIV) from chimpanzees to humans a century ago (Gao et al., 1999). Subsequent to this, HIV-1 Group M diversified in Central Africa into multiple distinct subtypes: A, B, C, D, F, G, H, J, K, and L (Robertson et al., 2000; Worobey et al., 2008; Yamaguchi et al., 2020). The global spread of HIV throughout the second half of the twentieth century led to the differential global distribution of HIV-1 subtypes (Tebit and Arts, 2011; Hemelaar, 2012; Faria et al., 2014).

Genetic divergence between HIV-1 subtypes is substantial, with Env displaying a median difference of 25% (range 20–36%) at the amino acid level between strains from different subtypes, whereas the difference is 17% (15–22%) for Gag (Korber et al., 2001). Recombination between different HIV strains has led to further diversification of the HIV pandemic (Hemelaar, 2013). Recombinant forms are classified as circulating recombinant forms (CRFs) if they are found in three or more epidemiologically distinct individuals or unique recombinant forms (URFs) if there is no evidence of onward transmission (Robertson et al., 2000). To date, 106 distinct CRFs have been identified (Zhou et al., 2020), and collectively, these CRFs have been estimated to account for 16.7% of HIV-1 infections worldwide (Hemelaar et al., 2019).

The immune response to HIV is multifaceted, with antibodies mainly directed against the envelope component of the virus, whereas cytotoxic T lymphocyte responses are preferentially directed against Gag and/or Pol (Goulder et al., 2000). The large genetic divergence between HIV-1 subtypes makes it difficult to elicit immune responses that are sufficiently cross-reactive between HIV-1 subtypes (Korber et al., 2001). Given the variation between HIV-1 subtypes, it has been a common approach in HIV vaccine design to match the HIV-1 subtype(s) of the immunogen in the candidate vaccine to the HIV-1 subtype(s) circulating in the target population. To date, a number of different vaccine concepts, each using immunogen HIV-1 subtype(s) matched to circulating HIV-1 subtype(s), have been tested in large-scale efficacy trials.

Firstly, recombinant HIV-1 envelope proteins were used as immunogens aimed at generating broadly neutralizing antibodies. A bivalent subtype B/B recombinant glycoprotein gp120 vaccine was trialed in North America and The Netherlands, where subtype B dominates, and a bivalent subtype B/E recombinant gp120 vaccine was tested in Thailand, where subtype B and CRF01_AE cocirculate. Neither of these vaccines proved efficacious (HIV Vaccine Study Group et al., 2005; Pitisuttithum et al., 2006).

Next, viral vectors were used with the aim of eliciting cytotoxic T lymphocyte responses. The first such vaccine consisted of adenovirus type-5 (Ad5) vectors expressing subtype B Gag, Pol, and Nef proteins. This vaccine was tested in North America, the Caribbean, South America, and Australia, where subtype B is the predominant HIV-1 subtype, and in South Africa, where subtype C dominates. In both trials, the vaccine did not prevent HIV-1 infection or lower the viral-load setpoint (Buchbinder et al., 2008; Gray et al., 2011).

A subsequent approach aimed to elicit both antibody and T-cell responses. This strategy consisted of priming with DNA plasmids expressing subtype B Gag, Pol, and Nef and subtypes A, B, and C Env proteins, followed by a boost consisting of Ad5 vectors expressing a subtype B Gag-Pol fusion protein and Env glycoproteins of subtypes A, B, and C. When tested in MSM populations in the United States (mainly subtype B), the vaccine did not reduce the rate of HIV-1 acquisition or the viral-load set point (Hammer et al., 2013).

A further attempt aimed at eliciting both humoral and cell-mediated immunity used a combination of a canarypox vector expressing subtype B Env gp41TM, Gag and Pol and CRF01_AE Env gp120 followed by a boost with bivalent subtype B/E recombinant gp120 proteins, chosen to match the B and CRF01_AE strains circulating in Thailand. The RV144 trial showed modest efficacy of this vaccine (Rerks-Ngarm et al., 2009). This vaccine concept was then adapted for use in South Africa by replacing the B/CRF01_AE immunogens with subtype C immunogens to match HIV-1 subtype C endemic in South Africa (Bekker et al., 2018). However, the trial of this vaccine was recently halted due to lack of efficacy (Adepoju, 2020).

Given the genetic divergence between HIV-1 subtypes and their differential global spread, it may be necessary to employ subtype-specific HIV-1 vaccines in individual countries according to their HIV-1 subtype distribution. To aid prioritization of HIV-1 subtypes for vaccine development, we aimed to estimate the global and regional need for therapeutic and prophylactic vaccines specific for different HIV-1 subtypes, taking account of the proportions of different HIV-1 variants circulating in each country, the genetic composition of HIV-1 recombinants, and the different genome segments of HIV that may be incorporated into a vaccine.

## Materials and Methods

### HIV-1 Molecular Epidemiology Data

Country-level HIV-1 molecular epidemiology data was collected by conducting a global survey and a comprehensive systematic review, as described previously (Hemelaar et al., 2019; Hemelaar et al., 2020). In total, 2,203 datasets with 383,519 samples were obtained from 116 countries across 1990–2015, with the data analyzed for four time periods: 2010–2015, 2005–2009, 2000–2004, and 1990–1999. In the current study, we used data from the latest time period (2010–2015) for analysis for most countries. For the small number of countries for which no data for 2010–2015 was available, data from the next most recent time period (2005–2009, 2000–2004, or 1990–1999) was used. These latter countries, as well as the time period for which data was used, are listed in Supplementary Material, p. 7.

### Reassignment of CRFs to “Pure” HIV-1 Subtypes

Given the large number of different CRFs (106 distinct CRFs identified to date (Los Alamos National Laboratory; Zhou et al., 2020), it would be impractical to make a vaccine specific for each CRF. Hence, we determined which “pure” HIV-1 subtypes contributed most to each CRF, both to each genome segment (*gag*, *pol*, *env*) as well as the full-length genome (which also includes accessory genes (*vif*, *vpu*, *vpr*, *nef*), the regulatory genes (*rev*, *tat*), and the 5′ and 3′ long terminal repeat regions). Information on the genetic composition of individual CRFs was obtained from the Los Alamos National Laboratory (LANL) website (Los Alamos National Laboratory). CRFs were reassigned to “pure” HIV-1 subtypes according to the HIV-1 subtype making the largest contribution to the full-length genome as well as each genome segment. In situations where unclassified sequences made the largest contribution, the next largest contributing HIV-1 subtype was taken. If the subtype composition of certain genome regions was unclear from the LANL website, the original paper describing the CRF was examined. The full list of CRFs and their reassignment to the “pure” HIV-1 subtypes, for full length as well as each genome segment, can be found in Supplementary Material, pp. 4–6. Unfortunately, reassignment could not be performed for CRF30_0206, CRF75_01B, CRF77_cpx, CRF79_0107, CRF80_0107, CRF81_cpx, and CRF84_A1D due to lack of data on their subtype composition.

### HIV-1 Subtype Proportions in Countries After Reassignment of CRFs to “Pure” HIV-1 Subtypes

Upon completion of the reassignment of CRFs to “pure” HIV-1 subtypes, the proportions of infections accounted for by each CRF in each country, as previously estimated (Hemelaar et al., 2020), were added to those of the relevant “pure” HIV-1 subtypes, thereby generating new proportions of infections that could be ascribed to each “pure” HIV-1 subtype for the full-length genome and each genome segment. Country-level “pure” HIV-1 subtype distributions were combined with UNAIDS data on the number of people living with HIV in each country in 2016 (UNAIDS, 2017) to generate estimates of regional and global “pure” HIV-1 subtype proportions (Supplementary Material, p. 2).

### Estimation of Numbers of Doses for Subtype-Specific Therapeutic and Prophylactic HIV-1 Vaccines

Upon estimation of the proportions of “pure” HIV-1 subtypes in each country, estimates for the numbers of doses needed for either therapeutic or prophylactic subtype-specific HIV-1 vaccines were calculated. Calculations were performed for three different scenarios, each using a different cut-off for HIV-1 subtypes eligible for inclusion in vaccines for each country: (1) Vaccinating against only the most common subtype circulating in each country (“most common subtype” scenario), (2) Vaccinating against subtypes with a prevalence of >5% in people living with HIV in each country (“>5% prevalence” scenario), and (3) Vaccinating against all circulating subtypes in each country (“all circulating subtypes” scenario). A fourth scenario was assessed for therapeutic vaccines, in which each HIV-infected individual would be vaccinated with a vaccine based on the HIV-1 subtype they were infected with. All calculations for each scenario were conducted for the full-length genome and each genome region (*gag*, *pol*, *env*).

For therapeutic vaccines, the target population was all people living with HIV in 2016, as estimated by UNAIDS (UNAIDS, 2017). For prophylactic vaccines, the target population was 10–49-year-old men and women, chosen to include most of the sexually active population as well as other high-risk groups, using estimates of population numbers in 2015 reported by the United Nations (United Nations, 2017). For both the therapeutic and prophylactic HIV-1 vaccine analyses, the entire target population in each country was to be vaccinated against every subtype that made the cut-off in each scenario, i.e., the most common subtype, all subtypes which contributed >5% of HIV infections, or all circulating subtypes. The estimated number of subtype-specific HIV-1 vaccine doses per country were subsequently aggregated at both the regional and global levels.

The term “dose” in this study was used to describe a “course of vaccination” with a subtype-specific HIV-1 vaccine. It may, however, be that a course of vaccination may consist of multiple doses of the same vaccine or a combination of different types of vaccines in a “prime-boost” configuration. All calculations were performed in Microsoft Excel.

## Results

### Global and Regional Distribution of HIV-1 Subtypes

**FIGURE 1:**
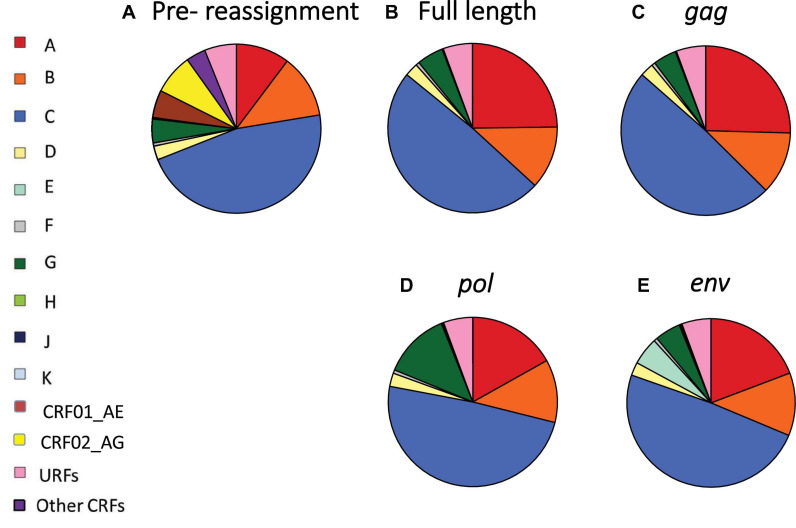
Global distribution of HIV-1 variants before and after reassignment of CRFs to “pure” HIV-1 subtypes. **(A)** Global proportions of HIV-1 subtypes A–K, CRF01_AE, CRF02_AG, other CRFs and URFs, based on most recent data available for each country. **(B–E)** Global proportions of HIV-1 variants after reassignment of CRFs to “pure” HIV-1 subtypes, based on full-length sequence **(B)**, *gag* **(C)**, *pol* **(D)**, and *env* **(E)**. CRF, circulating recombinant form; URF, unique recombinant form. Data underlying this figure is displayed in Supplementary Material, pp. 8–10.

**FIGURE 2:**
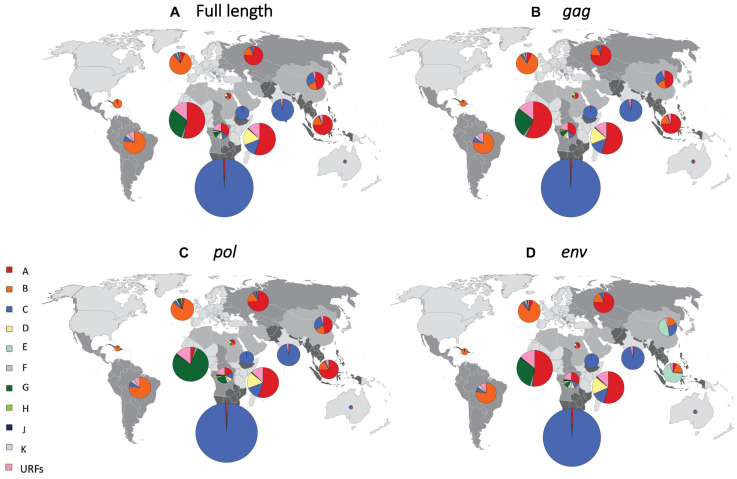
Regional distribution of HIV-1 subtypes after reassignment of CRFs to “pure” HIV-1 subtypes. Regional proportions of HIV-1 subtypes after reassignment of CRFs to “pure” HIV-1 subtypes, based on full-length sequence **(A)**, *gag* **(B)**, *pol* **(C)**, and *env* **(D)**. We grouped all countries into 14 regions, as previously described (Hemelaar et al., 2019). Individual regions are shaded differently on the world map. The proportion of HIV infections attributed to each subtype in each region is shown in pie charts superimposed onto the regions. The sizes (surface area) of the pie charts for each region are proportional to the relative number of people living with HIV in each region. URF, unique recombinant form. Data underlying this figure is displayed in Supplementary Material, pp. 8–10.

Given the difficulty in generating a vaccine for each individual HIV-1 recombinant, we first reassigned each CRF to a “pure” HIV-1 subtype (A-K) according to the HIV-1 subtype that contributed most to each CRF, both for the full length genome and the *gag, pol*, and *env* regions (Supplementary Material, pp. 4–6). Following reassignment of CRFs, we determined the global and regional proportions of infections caused by each of the “pure” HIV-1 subtypes, based on the most recent available HIV-1 subtype distribution data for each country (Figures 1, 2 and Supplementary Material, pp. 8–10).

After reassignment of CRFs based on the full-length genome, half of the global HIV infections were attributable to subtype C (49.1%), followed by subtype A (24.8%), B (12.0%), G (5.1%), D (2.5%), F (0.7%), and H, J, and K (0.1% each) (Figure 1B and Supplementary Material, pp. 8–10). Major changes in global HIV-1 subtype distribution resulting from reassignment of CRFs to “pure” HIV-1 subtypes were driven by the major recombinants CRF01_AE and CRF02_AG, as CRF01_AE is composed of subtype A in *gag* and *pol*, but subtype E in *env*, whereas CRF02_AG is composed of subtype A in *gag* and *env* and subtype G in *pol* (Figure 1 and Supplementary Material, pp. 4–6 and 8–10).

Subtype A contributed 25.4% of global HIV infections after CRF reassignment based on *gag*, 19.2% for *env*, and 16.9% for *pol* (Figures 1C–E and Supplementary Material, pp. 9–10). Subtype E contributed 5.3% of global infections after reassignment based on *env*, but none for *gag* and *pol*, as subtype E has never been identified for those genome segments. Subtype G constituted 12.7% of infections after reassignment based on *pol*, but only 5.0% for *env* and 4.4% for *gag*. The global contributions of other subtypes remained relatively stable following CRF reassignment (Figure 1 and Supplementary Material, pp. 8–10).

In South-East Asia, where CRF01_AE plays an important role, the proportion of infections attributable to subtype A was 74.4% after CRF reassignment based on the full-length analysis (Figure 2A and Supplementary Material, pp. 8–10). Subtype A also constituted 74.4 and 74.2% of infections for *gag* and *pol* in this region, whereas subtype E constituted 67.8% for *env* (Figures 2B–D and Supplementary Material, pp. 8–10). In East Asia, subtype A constituted 47.0% of infections for full length and *gag*, and 46.7% for *pol*. However, subtype E constituted 46.8% of infections for *env* in this region. In West Africa, where CRF02_AG plays a major role, subtype A constituted 52.6% of infections for full length and similar percentages for *gag* and *env*. However, subtype G constituted 78.7% for *pol*. In the other regions, which had fewer CRF infections, there was less change in HIV-1 subtype proportions following reassignment of CRFs to “pure” HIV-1 subtypes (Figure 2 and Supplementary Material, pp. 8–10).

### Therapeutic HIV-1 Vaccines

**FIGURE 3:**
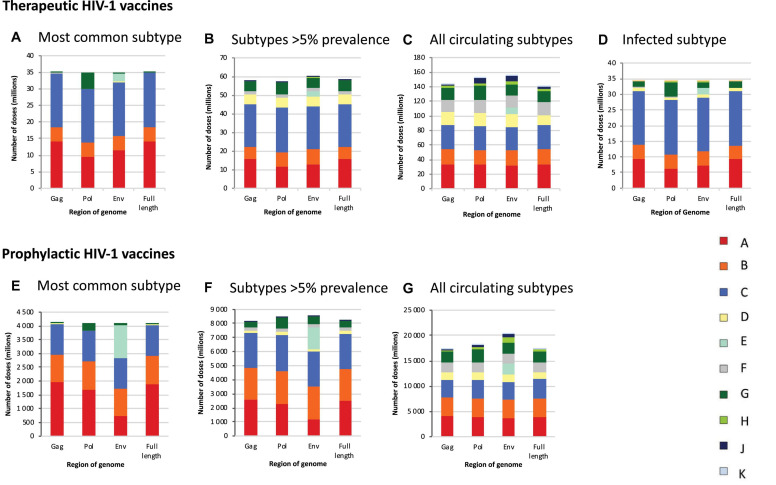
Global estimates of the number of doses of subtype-specific therapeutic and prophylactic HIV-1 vaccines. Estimates of the number of doses of subtype-specific therapeutic **(A–D)** and prophylactic **(E–G)** HIV-1 vaccines. Estimates are stratified according to genome region (*gag*, *pol*, *env*, and full length) and the number of subtypes against which people in each country are vaccinated, according to different scenarios, i.e., vaccinating people against the most common subtype in each country **(A,E)**, subtypes with a prevalence of >5% in people living with HIV in each country **(B,F)**, all subtypes circulating in each country **(C,G)**, and the HIV-1 subtype with which HIV-positive people are infected **(D)**. Data underlying this figure is displayed in Table 1.

**FIGURE 4:**
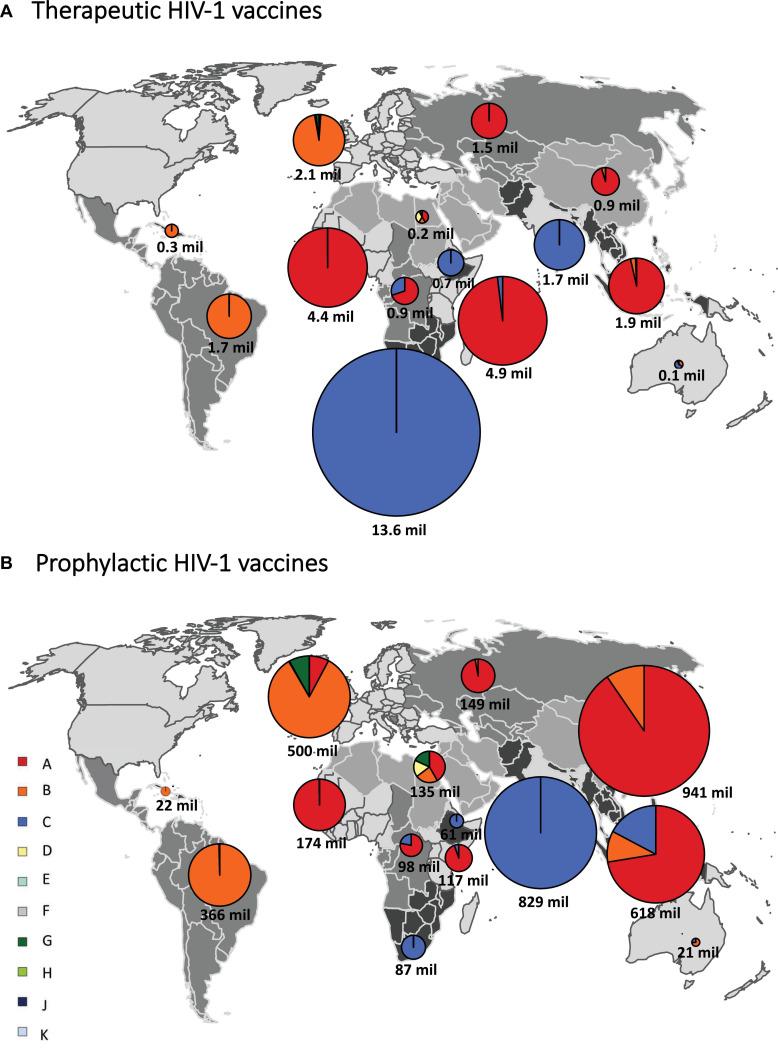
Regional estimates of the number of doses of subtype-specific therapeutic and prophylactic HIV-1 vaccines. Regional estimates for therapeutic **(A)** and prophylactic **(B)** HIV-1 vaccines based on the most common subtype, based on the full-length sequence, in each country. We grouped all countries into 14 regions, as previously described (Hemelaar et al., 2019). Individual regions are shaded differently on the world map. The estimates for vaccines based on each HIV-1 subtype are shown in pie charts superimposed onto the regions. The sizes of the pie charts (surface area) correspond to the relative number of vaccine doses for each region and the total number of vaccine doses is shown below each pie chart. mil, million. Data underlying this figure is displayed in Supplementary Material, p. 11.

If HIV-infected people would be vaccinated against the most common subtype circulating in each country (“most common subtype” scenario), based on the full-length genome, 35.1 million vaccine doses would be required globally, of which 16.5 million were subtype C, 14.3 million subtype A, and 4.2 million subtype B, with much fewer doses for other subtypes (Figure 3A and Table 1). A vaccine based on *env* required 2.6 million subtype E doses, and a vaccine based on *pol* required 4.8 million subtype G doses (Figure 3A and Table 1). The global need for a therapeutic subtype C vaccine was largely driven by Southern Africa and South Asia (Figure 4A and Supplementary Material, p. 11). The need for a subtype A vaccine was largely driven by East and West Africa, as well as South-East Asia and Eastern Europe and Central Asia. Finally, the global need for a subtype B vaccine was driven by Western and Central Europe and North America and Latin America (Figure 4A and Supplementary Material, p. 11).

**TABLE 1:** Global estimates of numbers of doses (millions) of subtype-specific therapeutic and prophylactic HIV-1 vaccines.

			**HIV-1 Subtypes**										
**Vaccine Type**	**Scenario**	**Genome Region**	**A**	**B**	**C**	**D**	**E**	**F**	**G**	**H**	**J**	**K**	**Total**
Therapeutic	Most common	*gag*	14.28	4.21	16.21	0.06	0.00	0.02	0.04	0.00	0.00	0.00	34.81
		*pol*	9.54	4.22	16.21	0.06	0.00	0.02	4.78	0.00	0.00	0.00	34.81
		*env*	11.65	4.23	16.21	0.06	2.60	0.00	0.06	0.00	0.00	0.00	34.81
		Full length	14.25	4.22	16.50	0.06	0.00	0.02	0.06	0.00	0.00	0.00	35.10
	>5%	*gag*	15.94	6.47	22.73	5.34	0.00	1.62	5.11	0.28	0.20	0.00	57.69
		*pol*	11.67	7.75	23.89	5.34	0.00	1.62	6.53	0.28	0.32	0.00	57.38
		*env*	13.00	7.75	23.14	5.28	2.92	1.62	5.70	0.31	0.32	0.00	60.02
		Full length	15.94	6.52	22.74	5.28	0.00	1.63	5.63	0.28	0.20	0.00	58.24
	All subtypes	*gag*	33.12	20.78	32.73	18.11	0.00	17.82	15.20	3.14	2.27	0.70	143.89
		*pol*	32.71	20.78	32.73	18.11	0.00	17.89	18.70	3.05	8.05	1.09	153.12
		*env*	31.20	20.89	32.11	17.96	8.54	17.81	14.22	4.02	8.10	1.09	155.94
		Full length	32.86	20.78	33.31	14.08	0.00	17.69	15.66	3.14	2.68	1.07	141.27
	Infected subtype	*gag*	9.50	4.34	17.33	1.03	0.00	0.25	1.62	0.04	0.04	0.02	34.17
		*pol*	6.37	4.36	17.33	1.03	0.00	0.24	4.66	0.04	0.12	0.02	34.17
		*env*	7.33	4.38	17.33	1.02	1.85	0.25	1.81	0.07	0.12	0.02	34.17
		Full length	9.26	4.36	17.33	1.02	0.00	0.25	1.85	0.04	0.04	0.02	34.17
Prophylactic	Most common	*gag*	1,968.11	1,004.82	1,095.35	23.24	0.00	2.97	1.34	0.00	0.00	0.00	4,095.84
		*pol*	1,702.24	1,010.52	1,095.35	23.24	0.00	2.97	261.52	0.00	0.00	0.00	4,095.84
		*env*	718.02	1,012.00	1,095.35	23.24	1,180.43	1.50	65.31	0.00	0.00	0.00	4,095.84
		Full length	1,898.45	1,010.52	1,116.87	23.24	0.00	2.97	65.31	0.00	0.00	0.00	4,117.36
	>5%	*gag*	2,561.34	2,256.68	2,486.53	203.94	0.00	253.16	356.69	21.52	8.40	0.00	8,148.27
		*pol*	2,298.97	2,345.43	2,567.32	203.94	0.00	253.16	748.03	21.52	19.02	0.00	8,457.38
		*env*	1,160.36	2,345.43	2,481.26	196.47	1,557.26	253.16	489.63	27.85	19.02	0.00	8,530.44
		Full length	2,522.69	2,269.40	2,491.46	180.70	0.00	258.09	477.17	21.52	8.40	0.00	8,229.43
	All subtypes	*gag*	4,033.34	3,627.84	3,660.23	1,443.14	2.46	1,952.32	2,096.18	349.77	84.54	71.15	17,320.97
		*pol*	3,907.84	3,627.84	3,660.23	1,443.14	2.46	2,007.39	2,685.46	345.61	457.35	158.67	18,296.00
		*env*	3,643.27	3,637.83	3,604.44	1,407.96	2,112.43	1,950.97	2,190.60	1,200.43	457.57	157.98	20,363.47
		Full length	3,942.57	3,627.84	3,814.19	1,274.02	2.46	1,945.75	2,243.83	362.80	151.96	129.63	17,495.05

*Estimates are stratified according to genome region (gag, pol, env, and full length) and the number of subtypes against which people in each country are vaccinated, according to different scenarios, i.e., vaccinating people against the most common subtype in each country (“most common”), subtypes with a prevalence of > 5% in people living with HIV in each country (“>5%”), all subtypes circulating in each country (“all subtypes”), and the HIV-1 subtype with which HIV-positive people are infected (infected subtype).*

If HIV-infected people would be vaccinated against subtypes with a prevalence of >5% in people living with HIV in each country (“>5% prevalence” scenario), based on the full-length genome, 58.2 million vaccine doses were estimated, of which 22.7 million would be subtype C, 15.9 million subtype A, 6.5 million subtype B, 5.6 million subtype G, and 5.3 million subtype D (Figure 3B and Table 1). A vaccine based on *env* required 2.9 million subtype E doses.

If HIV-infected people would be vaccinated against all circulating subtypes in each country (“all circulating subtypes” scenario), 141.3 million doses of vaccine would be required, based on the full-length genome, of which 33.3 million would be subtype C, 32.9 million subtype A, 20.8 million subtype B, 17.7 million subtype F, 15.7 million subtype G, 14.1 million subtype D, 3.1 million subtype H, 2.7 million subtype J and 1.1 million subtype K (Figure 3C and Table 1). A vaccine based on *env* required 8.5 million subtype E doses.

In the final scenario in which each infected individual would be vaccinated only against the subtype with which they are already infected, 34.2 million vaccine doses were estimated, based on the full-length genome, of which 17.3 million would be subtype C, 9.3 million subtype A, 4.4 million subtype B, 1.9 million subtype G, and 1.0 million subtype D, reflecting the global distribution of HIV-1 variants (Figures 1, 3D, Table 1, and Supplementary Material, 8–10).

#### Prophylactic HIV-1 Vaccines

If all 10–49-year-old people would be vaccinated against the most common subtype circulating in each country (“most common subtype” scenario), based on the full-length genome, an estimated 4.1 billion doses of vaccines would be required globally, of which 1.9 billion were subtype A, 1.1 billion subtype C, and 1.0 billion subtype B (Figure 3E and Table 1). A vaccine based on *env* required 1.2 billion subtype E doses, and a vaccine based on *pol* required 262 million subtype G doses. The global need for a prophylactic subtype A vaccine was largely driven by East Asia and South-East Asia (Figure 4B and Supplementary Material, p. 11). This was due to their large populations as well as the endemic nature of CRF01_AE in these regions. The need for a subtype C vaccine was driven largely by South Asia, and the need for a subtype B vaccine was driven by Western and Central Europe and North America and Latin America.

In the “>5% prevalence” scenario, 8.2 billion doses of a vaccine based on the full-length genome were estimated, of which 2.5 billion would be subtype A, 2.5 billion subtype C, and 2.3 billion subtype B (Figure 3F and Table 1). A vaccine based on *env* required 1.6 billion subtype E doses.

Finally, in the “all circulating subtypes” scenario, for a vaccine based on the full-length genome, 17.5 billion vaccine doses would be required, of which 3.9 billion would be subtype A, 3.8 billion subtype C, 3.6 billion subtype B, 2.2 billion subtype G, 1.9 billion subtype F, 1.3 billion subtype D, and 0.4 billion for subtype H (Figure 3G and Table 1). A vaccine based on *env* required 2.1 billion subtype E doses.

## Discussion

In this study, we estimated the global and regional need for subtype-specific therapeutic and prophylactic HIV-1 vaccines. When targeting the most common HIV-1 subtype in each country, we estimated the largest number of therapeutic vaccine doses were needed for a subtype C vaccine (16.5 million), followed by subtype A (14.3 million) and subtype B (4.2 million). A vaccine based on *env* required 2.6 million subtype E doses, and a vaccine based on *pol* required 4.8 million subtype G doses. The need for therapeutic subtype C vaccines was largely driven by the endemicity of subtype C in Southern Africa and South Asia, the need for subtype A vaccines by East and West Africa, and the need for subtype B vaccines by Western and Central Europe and North America and Latin America.

For prophylactic vaccines targeting the most common HIV-1 subtype in each country, 1.9 billion doses of subtype A vaccine were estimated, followed by subtype C (1.1 billion) and subtype B (1.0 billion). A vaccine based on *env* required 1.2 billion subtype E doses, and a vaccine based on *pol* required 0.3 billion subtype G doses. The need for prophylactic subtypes A and E vaccines was largely driven by East Asia and South-East Asia, owing to their large populations as well as prevalence of CRF01_AE. The need for a prophylactic subtype C vaccine was largely driven by South Asia.

Employing vaccines against more than one HIV-1 subtype in each country, as estimated in the “>5% prevalence” and “all circulating subtypes” scenarios, dramatically increases the number of vaccine doses and number of different subtype-specific vaccines required for both therapeutic and prophylactic vaccines.

It is apparent that to provide global coverage against the most common HIV-1 subtype circulating in each country, subtype-specific therapeutic and prophylactic HIV-1 vaccines need to be directed against subtypes A, B, and C. Vaccines targeting the envelope protein would also need to include subtype E and those targeting Pol need to include subtype G. If subtype-specific vaccines are also directed against less common HIV-1 subtypes in each country, vaccines targeting subtypes D, F, H, and K also need to be considered and would require up to five times more vaccine doses in total.

This study has several strengths. We utilized the largest available global HIV-1 molecular epidemiology database and applied the novel approach of reassigning CRFs to “pure” HIV-1 subtypes based on their genetic composition. This enabled us to address the complexity posed by multiple distinct recombinants and to generate new estimates of the proportion of infections caused by each “pure” HIV-1 subtype. In addition, because HIV-1 vaccines could aim to elicit antibodies or T-cell responses or both, we generated estimates for subtype-specific HIV-1 vaccines based on *env*, *gag*, and *pol* as well as the full-length genome. Moreover, we conducted separate analyses for therapeutic and prophylactic vaccines. In addition, we examined a number of different scenarios according to the number of HIV-1 subtypes eligible for inclusion in a vaccine.

Our study also has some limitations. Although CRFs were reassigned to “pure” HIV-1 subtypes based on their genetic composition, we do not know the extent of their overlapping immunogenic properties. Although the target population for a therapeutic vaccine are people living with HIV, the target population for a prophylactic vaccine is less certain. We opted to estimate a one-off vaccination of all people aged 10–49 years old, to include most sexually active people and other risk groups (Marzetta et al., 2010). We made no distinction between routine and catch-up vaccinations (Marzetta et al., 2010). This comprehensive approach may have led to higher estimates of numbers of doses needed, but did allow us to gauge the relative importance of HIV-1 subtypes for regional and global vaccine development to enable prioritization of relevant HIV-1 subtypes. Vaccine efficacy may differ between subtype-specific vaccines and was not factored in Dimitrov et al. (2015). Moreover, a putative vaccine with high efficacy would likely be administered to larger populations whereas a vaccine with low/moderate efficacy would more likely be limited to high risk groups (Esparza et al., 2003). Duration of protection offered by a putative vaccine was not modeled and consequently revaccination was also not factored in. Furthermore, we aimed to estimate the need for subtype-specific vaccines and did not estimate the actual uptake or use, which depends on factors such as adoption time, accessibility and acceptability, which will vary by country (Marzetta et al., 2010). Lastly, cost and cost-effectiveness were not taken into account.

There are also limitations to the concept of subtype-specific HIV-1 vaccines. One issue is the need to generate multiple different vaccines for the different HIV-1 subtypes. This could be partially overcome by formulation of multivalent vaccines (“cocktails”) incorporating multiple subtype-specific preparations (Korber et al., 2017). Another limitation is that a subtype-specific HIV-1 vaccine would need to be matched to locally circulating strains, which requires availability of up-to-date HIV-1 diversity data and the relevant subtype-specific HIV-1 vaccines. Furthermore, protection would be limited to a certain geographical region and thereby limit travel to other regions, while at the same time leave vaccinated people vulnerable to infection by newly imported strains of HIV, as HIV-1 subtype distribution is very dynamic (Hemelaar et al., 2019).

A crucial outstanding limitation of subtype-specific HIV-1 vaccines is the issue of intrasubtype genetic diversity (Korber et al., 2001; Gaschen et al., 2002). The HIV-1 vaccine recently tested, and proven ineffective, in South Africa consisted of a recombinant canarypox vaccine, which contained a subtype C *env* gp120 isolate sequence (96ZM651 from Zambia), and a bivalent subtype C gp120 consisting of two distinct subtype C recombinant monomeric Env gp120 proteins (derived from isolates TV1.C from South Africa and 1086.C from Malawi) (Zambonelli et al., 2016; Bekker et al., 2018). Utilization of subtype C isolate sequences in the vaccine, matching subtype C dominating in South Africa, was hoped to lead to protective immunogenicity. However, intrasubtype diversity is considerable, with median percentage amino acid differences within HIV-1 subtypes estimated at 17% (range 4–30%) for Env and 8% (2–15%) for Gag (Korber et al., 2001), thereby limiting the potential for eliciting cross-reactive protective immune responses. One way to reduce the genetic distance between vaccine immunogens and circulating strains is the inclusion of artificial centralized sequences, such as consensus, ancestral or center-of tree sequences (Gaschen et al., 2002; Nickle et al., 2003). For example, subtype C isolate sequences are around 5–15% different to other subtype C isolate sequences, whereas, a subtype C consensus amino acid sequence is only around 3–8% different from individual subtype C isolates (Gaschen et al., 2002). Of note, a group M consensus sequence (i.e., a consensus of all subtype consensus sequences) would be around 5–15% different to individual circulating HIV-1 isolates and therefore not better than a subtype-matched isolate sequence (Gaschen et al., 2002). Indeed, the use of isolate sequences in all candidate HIV vaccines tested in phase 3 trials to date may have limited cross-reactivity and hence limited efficacy (HIV Vaccine Study Group et al., 2005; Pitisuttithum et al., 2006; Buchbinder et al., 2008; Rerks-Ngarm et al., 2009; Gray et al., 2011; Hammer et al., 2013; Bekker et al., 2018). Thus, the use of subtype consensus (or ancestral or center-of-tree) sequences may be a more successful approach in the future.

Ideally, a globally effective HIV vaccine will need to confer protection against all diverse HIV-1 subtypes and recombinants. There are multiple HIV vaccine efforts on-going, utilizing a number of different approaches to address HIV-1 diversity. One approach is to use mosaic vaccines, which have been shown to elicit increased breadth and depth of immune responses (Barouch et al., 2010). The HVTN 705/Imbokodo trial currently underway in southern Africa is evaluating a tetravalent vaccine composed of adenovirus serotype 26 vector expressing mosaic *gag, pol* and *env* inserts combined with subtype C gp140 Env protein, with the intention of eliciting responses against a wide range of HIV subtypes, but still matching subtype C predominant in the region. The HVTN 706/Mosaico trial taking place in North America, Western Europe, and Latin America evaluates a nearly identical mosaic vaccine, which also includes a mosaic gp140 glycoprotein (Baden et al., 2020). Other approaches in preclinical development include focussing on conserved or structurally important regions of HIV (Letourneau et al., 2007; Gaiha et al., 2019). For all HIV-1 vaccine approaches, it is crucial to have up-to-date knowledge of HIV-1 genetic diversity to allow prioritization and development of vaccine concepts that are likely to provide the greatest benefit to specific countries, regions, and the world.

## Data Availability Statement

The original contributions presented in the study are included in the article/Supplementary Material, further inquiries can be directed to the corresponding author.

## Ethics Statement

The studies involving human participants were reviewed and approved by local ethics committees of contributing studies. The participants provided their written informed consent to participate in this study.

## Author Contributions

RE and MJ conducted the analyses, prepared the figures and tables, interpreted the data, and wrote the first draft of the manuscript. RE, JY, LD-T, SK, and JH collected the data. JH conceived, designed and coordinated the study, designed the analysis and figures, interpreted the data, and wrote the manuscript. All authors read and approved the final version of the manuscript.

## Conflict of Interest

The authors declare that the research was conducted in the absence of any commercial or financial relationships that could be construed as a potential conflict of interest.
