# Supplementary material for: Global and Regional Estimates for Subtype-Specific Therapeutic and Prophylactic HIV-1 Vaccines: A Modeling Study
Source: Front Microbiol. 2021 Jul 15;12:690647. doi: 10.3389/fmicb.2021.690647 (PMC8320730; doi:10.3389/fmicb.2021.690647)
Supplement: Supplementary file 1 [file Data_Sheet_1.PDF]

Global and regional estimates for subtype-specific therapeutic and prophylactic HIV-1 vaccines.

Appendix

Page

Supplementary Figure 1: Study flow diagram. 2

Supplementary Figure 2: Regional distribution of HIV-1 variants before reassignment of CRFs to 'pure' HIV-1 subtypes. 3

Supplementary Table 1: Reassignment of CRFs to 'pure' HIV-1 subtypes. 4-6

Supplementary Table 2: Time periods of country HIV-1 subtyping data. 7

Supplementary Table 3: Global and regional HIV-1 subtype distributions before and after reassignment of CRFs to 'pure' HIV-1 subtypes. 8-10

Supplementary Table 4: Regional and global estimates of number of doses of subtype-specific therapeutic and prophylactic HIV-1 vaccines. 11

WHO-UNAIDS Network for HIV Isolation and Characterisation 12-15

References 16

Supplementary Figure 1.

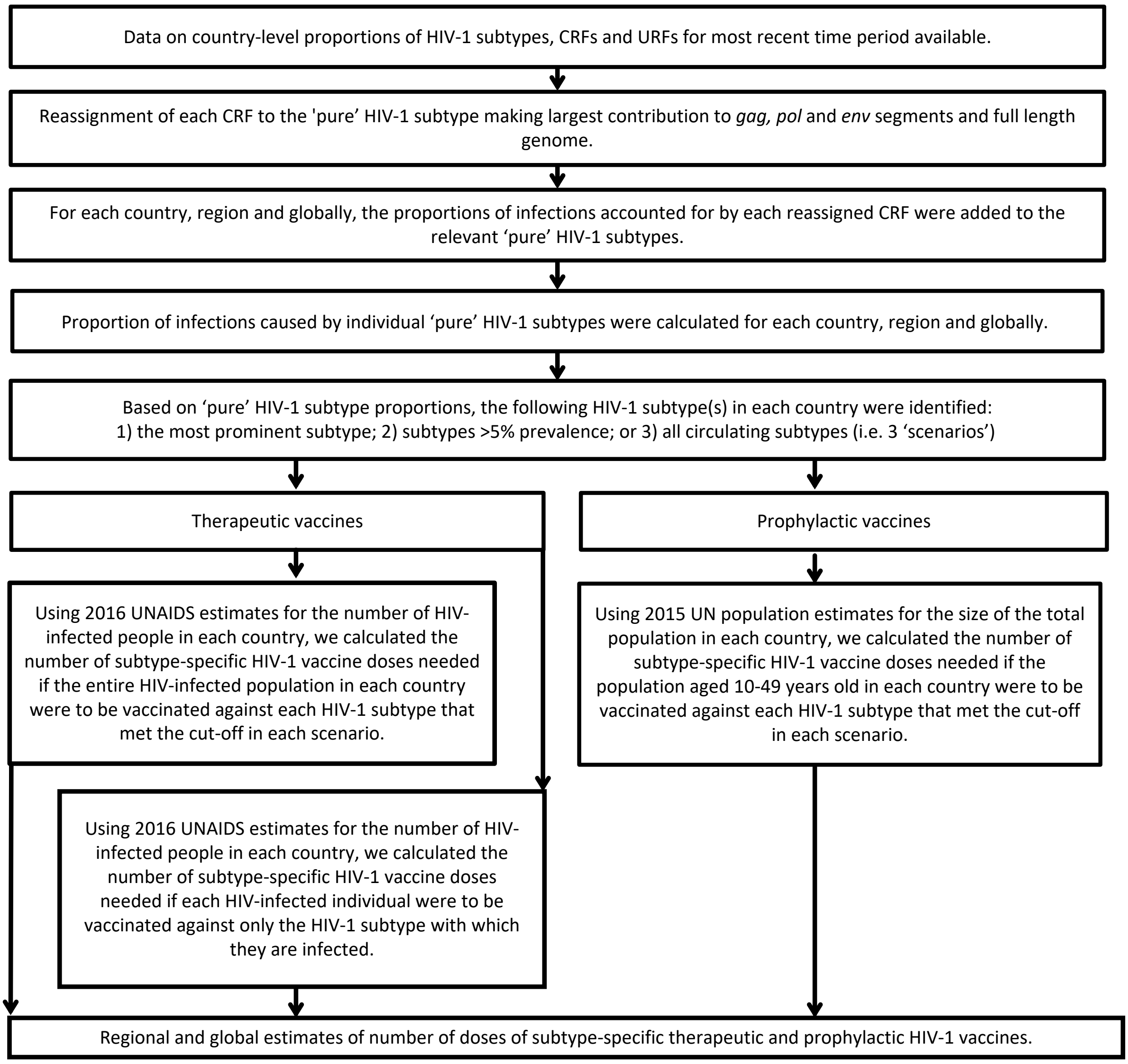

Supplementary Figure 1: Study flow diagram

Supplementary Figure 2.

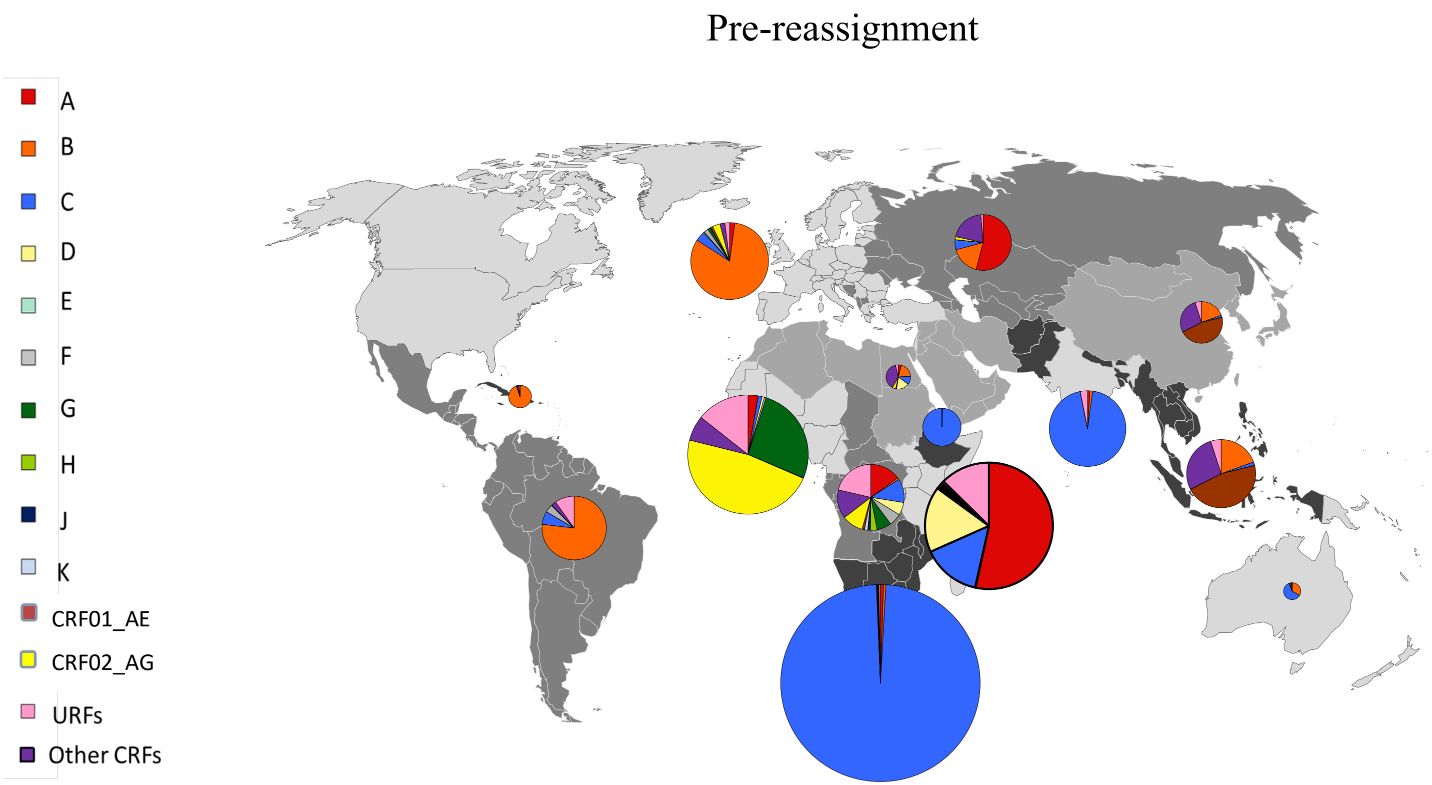

Supplementary Figure 2: Regional distribution of HIV-1 variants before reassignment of CRFs to ‘pure’ HIV-1 subtypes.

Supplementary Table 1.

	gag	*pol*	*env*	**full-length**
**CRF01_AE**	A	A	E	A
**CRF02_AG**	A	G	A	A
**CRF03_AB**	A	B	B	B
**CRF04_cpx (1)**	A	K	A	A
**CRF05_DF**	D	D	F	F
**CRF06_cpx**	A	G	G	G
**CRF07_BC**	C	C	C	C
**CRF08_BC**	C	C	C	C
**CRF09_cpx (2)**	A	G	A	A
**CRF10_CD**	D	D	D	D
**CRF11_cpx**	A	J	J	A
**CRF12_BF**	F	F	F	F
**CRF13_cpx**	G	G	A	G
**CRF14_BG**	G	G	B	G
**CRF15_01B**	CRF01_AE	CRF01_AE	B	CRF01_AE
**CRF16_A2D**	A2	A2	A2	A2
**CRF17_BF**	F	F	F	F
**CRF18_cpx**	A	G	H	A
**CRF19_cpx**	D	D	A	D
**CRF20_BG**	G	G	G	G
**CRF21_A2D (3)**	D	D	D	D
**CRF22_01A1**	CRF01_AE	A1	A1	A1
**CRF23_BG**	G	G	G	G
**CRF24_BG**	G	G	G	G
**CRF25_cpx (4)**	G	g	G	G
**CRF26_AU**	A	A	A	A
**CRF27_cpx**	G	J	CRF01_AE	G
**CRF28_BF**	F	B	B	B
**CRF29_BF**	F	F	B	B
**CRF30_0206**	n/a	n/a	n/a	n/a
**CRF31_BC**	C	C	C	C
**CRF32_06A1**	A	CRF06_cpx	CRF06_cpx	CRF06_cpx
**CRF33_01B**	CRF01_AE	CRF01_AE	CRF01_AE	CRF01_AE
**CRF34_01B**	CRF01_AE	CRF01_AE	CRF01_AE	CRF01_AE
**CRF35_AD**	A	A	A	A
**CRF36_cpx**	CRF01_AE	CRF02_AG	A	CRF02_AG
**CRF37_cpx**	A	A	A1	A
**CRF38_BF1**	F	F	F	F
**CRF39_BF**	B	B	F	B

**CRF40_BF**	F	F	F	F
**CRF41_CD**	D	D	D	D
**CRF42_BF**	F	B	B	B
**CRF43_02G**	G	CRF02_AG	G	G
**CRF44_BF**	F	F	F	F
**CRF45_AKU**	A	A	A	A
**CRF46_BF1**	F	F	F	F
**CRF47_BF (5)**	F	B	B	B
**CRF48_01B**	CRF01_AE	CRF01_AE	CRF01_AE	CRF01_AE
**CRF49_cpx**	A	C	J	J
**CRF50_A1D (6)**	D	A	D	A
**CRF51_01B**	B	B	CRF01_AE	B
**CRF52_01B**	CRF01_AE	CRF01_AE	B	CRF01_AE
**CRF53_01B**	CRF01_AE	CRF01_AE	CRF01_AE	CRF01_AE
**CRF54_01B**	B	B	CRF01_AE	B
**CRF55_01B**	CRF01_AE	CRF01_AE	CRF01_AE	CRF01_AE
**CRF56_cpx**	CRF02_AG	CRF02_AG	CRF02_AG	CRF02_AG
**CRF57_BC**	C	C	C	C
**CRF58_01B**	CRF01_AE	CRF01_AE	CRF01_AE	CRF01_AE
**CRF59_01B**	CRF01_AE	CRF01_AE	B	CRF01_AE
**CRF60_BC**	C	C	C	C
**CRF61_BC**	C	C	C	C
**CRF62_BC**	C	C	C	C
**CRF63_02A1**	CRF02_AG	CRF02_AG	CRF02_AG	CRF02_AG
**CRF64_BC**	C	C	C	C
**CRF65_cpx**	C	C	C	C
**CRF67_01B**	CRF01_AE	B	CRF01_AE	CRF01_AE
**CRF68_01B**	CRF01_AE	B	CRF01_AE	CRF01_AE
**CRF69_01B**	CRF01_AE	B	CRF01_AE	CRF01_AE
**CRF70_BF**	F	F	B	B
**CRF71_BF**	F	F	B	B
**CRF72_BF**	F	F	F	F
**CRF73_BG**	G	G	B	G
**CRF74_01B**	CRF01_AE	CRF01_AE	CRF01_AE	CRF01_AE
**CRF75_01B**	n/a	n/a	n/a	n/a
**CRF76_01B**	B	B	CRF1	B
**CRF77_cpx**	n/a	n/a	n/a	n/a
**CRF78_cpx**	CRF01_AE	CRF01_AE	CRF01_AE	CRF01_AE
**CRF79_0107**	n/a	n/a	n/a	n/a
**CRF80_0107**	n/a	n/a	n/a	n/a
**CRF81_cpx**	n/a	n/a	n/a	n/a
**CRF82_cpx**	C	C	C	C

**CRF83_cpx**	C	C	CRF01_AE	C
**CRF84_A1D**	n/a	n/a	n/a	n/a
**CRF85_BC**	C	C	C	C
**CRF86_BC**	C	B	C	C
**CRF87_cpx**	C	C	C	C
**CRF88_BC**	C	C	C	C

Supplementary Table 1: **Reassignment of CRFs to 'pure' HIV-1 subtypes.**
CRFs were reassigned to 'pure' HIV-1 subtypes based on the subtype which made the largest contribution to each genome segment (*gag, pol, env*) or the whole genome. Reassignment was performed using genome data in the Los Alamos National Laboratory database (LANL) (https://www.hiv.lanl.gov/content/sequence/HIV/CRFs/CRFs.html) or based on primary literature where indicated by references.
"n/a" indicates CRFs for which there was no sequencing/subtype data available in the LANL database or in the literature.

Supplementary Table 2.

Country	Time Period	Country	Time Period
Lebanon	1990-1999	Cape Verde	2005-2009
Moldova (Republic of)	1990-1999	Chile	2005-2009
Norway	1990-1999	Djibouti	2005-2009
Sudan	1990-1999	Fiji	2005-2009
Belarus	2000-2004	Gabon	2005-2009
Denmark	2000-2004	Georgia	2005-2009
Ecuador	2000-2004	Ghana	2005-2009
Luxembourg	2000-2004	Japan	2005-2009
New Zealand	2000-2004	Latvia	2005-2009
Oman	2000-2004	Lesotho	2005-2009
Papua New Guinea	2000-2004	Madagascar	2005-2009
Paraguay	2000-2004	Mongolia	2005-2009
Uzbekistan	2000-2004	Pakistan	2005-2009
Azerbaijan	2000-2004	Singapore	2005-2009
Austria	2005-2009	Slovakia	2005-2009
Bangladesh	2005-2009	Uruguay	2005-2009
Bolivia	2005-2009	Zambia	2005-2009

Supplementary Table 2. Time periods of country HIV-1 subtyping data.
The time periods from which HIV-1 subtyping data was used for each country are displayed. For all countries not listed, data from the most recent time period (2010-2015) was used.

Supplementary Table 3.

A. Pre-reassignment

Region	Proportion (%) of HIV-1 infections attributed to HIV-1 variants													
	A	B	C	D	E	F	G	H	J	K	URFs	CRF01_AE	CRF02_AG	Other CRFs
Caribbean	0.34	94.41	0.40	0.18	n/a	0.01	0.43	0.07	0.01	0.00	1.64	0.02	0.07	2.42
Latin America	0.09	76.78	6.64	0.02	n/a	4.13	0.00	0.02	0.00	0.00	9.81	0.00	0.10	2.42
Western and Central Europe and North America	2.16	82.02	4.11	0.35	n/a	1.92	1.18	0.03	0.01	0.03	1.98	0.96	3.23	2.03
Eastern Europe & Central Asia	54.03	16.80	5.79	0.01	n/a	0.00	0.01	0.00	0.00	0.00	1.36	0.00	1.72	20.28
South Asia	0.96	1.18	94.76	0.00	n/a	0.00	0.00	0.00	0.00	0.00	3.11	0.00	0.00	0.00
South-East Asia	6.26	17.68	1.77	0.23	n/a	0.00	0.14	0.00	0.00	0.00	5.55	66.37	0.23	1.76
East Asia	0.04	19.40	1.64	0.01	n/a	0.01	0.04	0.00	0.00	0.00	4.92	46.25	0.12	27.57
Oceania	0.63	32.71	61.15	0.10	n/a	0.15	0.19	0.00	0.00	0.00	0.60	3.68	0.56	0.23
Middle East & North Africa	3.98	20.17	11.03	17.18	n/a	0.19	0.32	0.00	0.00	0.00	3.74	0.66	4.31	38.43
West Africa	2.69	0.02	1.05	0.66	n/a	0.46	26.51	0.05	0.00	0.00	14.26	0.09	47.40	6.83
East Africa	53.59	0.25	14.32	16.58	n/a	0.08	0.49	0.00	0.50	0.00	12.64	0.61	0.17	0.78
Ethiopia	0.57	0.00	99.43	0.00	n/a	0.00	0.00	0.00	0.00	0.00	0.00	0.00	0.00	0.00
Central Africa	14.97	0.91	11.64	6.26	n/a	6.11	7.06	3.26	1.27	1.59	21.33	1.11	10.34	14.14
Southern Africa	0.50	0.38	98.47	0.06	n/a	0.04	0.10	0.00	0.01	0.00	0.23	0.00	0.09	0.10
Global	11.18	11.97	47.03	2.69	n/a	0.59	4.25	0.11	0.11	0.05	5.78	5.00	7.57	5.78

B. Full-length reassignment

Region	Proportion (%) of HIV-1 infections attributed to HIV-1 variants											
	A	B	C	D	E	F	G	H	J	K	URFs	Un-reassigned CRFs
Caribbean	0.89	94.41	0.40	1.26	0.00	0.03	1.18	0.07	0.01	0.00	1.64	0.11
Latin America	0.20	77.33	7.62	0.02	0.00	4.98	0.03	0.02	0.00	0.00	9.81	0.00
Western and Central Europe and North America	6.60	82.37	4.19	0.38	0.00	2.21	2.04	0.03	0.01	0.03	1.98	0.16
Eastern Europe & Central Asia	76.00	16.83	5.80	0.01	0.00	0.00	0.01	0.00	0.00	0.00	1.36	0.00
South Asia	0.96	1.18	94.76	0.00	0.00	0.00	0.00	0.00	0.00	0.00	3.11	0.00
South-East Asia	74.40	17.72	1.94	0.24	0.00	0.00	0.15	0.00	0.00	0.00	5.55	0.00
East Asia	47.02	19.40	28.60	0.01	0.00	0.01	0.04	0.00	0.00	0.00	4.92	0.00
Oceania	4.98	32.72	61.21	0.10	0.00	0.18	0.22	0.00	0.00	0.00	0.60	0.00
Middle East & North Africa	41.55	20.29	11.09	17.18	0.00	0.19	5.94	0.00	0.00	0.00	3.74	0.00
West Africa	52.61	0.02	1.05	0.67	0.00	0.63	30.71	0.05	0.01	0.00	14.26	0.00
East Africa	54.51	0.25	14.34	17.13	0.00	0.08	0.56	0.00	0.50	0.00	12.64	0.00
Ethiopia	0.57	0.00	99.43	0.00	0.00	0.00	0.00	0.00	0.00	0.00	0.00	0.00
Central Africa	35.09	1.11	11.69	6.61	0.00	6.19	11.85	3.26	1.27	1.59	21.33	0.00
Southern Africa	0.64	0.38	98.47	0.12	0.00	0.04	0.10	0.00	0.01	0.00	0.23	0.00
Global	24.79	12.02	49.06	2.54	0.00	0.67	5.05	0.11	0.09	0.05	5.62	0.01

C. *gag* reassignment

Region	Proportion (%) of HIV-1 infections attributed to HIV-1 variants											
	A	B	C	D	E	F	G	H	J	K	URFs	Un-reassigned CRFs
Caribbean	0.89	94.41	0.40	1.28	0.00	0.01	1.18	0.07	0.01	0.00	1.64	0.11
Latin America	0.49	76.87	7.62	0.06	0.00	5.14	0.00	0.02	0.00	0.00	9.81	0.00
Western and Central Europe and North America	7.13	82.02	4.19	0.38	0.00	2.49	1.57	0.03	0.01	0.03	1.98	0.16
Eastern Europe & Central Asia	76.02	16.80	5.80	0.01	0.00	0.00	0.01	0.00	0.00	0.00	1.36	0.00
South Asia	0.96	1.18	94.76	0.00	0.00	0.00	0.00	0.00	0.00	0.00	3.11	0.00
South-East Asia	74.41	17.72	1.94	0.24	0.00	0.00	0.15	0.00	0.00	0.00	5.55	0.00
East Asia	47.02	19.40	28.60	0.01	0.00	0.01	0.04	0.00	0.00	0.00	4.92	0.00
Oceania	5.00	32.71	61.21	0.10	0.00	0.18	0.20	0.00	0.00	0.00	0.60	0.00
Middle East & North Africa	47.16	20.17	11.09	17.18	0.00	0.32	0.34	0.00	0.00	0.00	3.74	0.00
West Africa	56.56	0.02	1.05	0.75	0.00	0.56	26.77	0.05	0.00	0.00	14.26	0.00
East Africa	54.48	0.25	14.34	17.22	0.00	0.08	0.49	0.00	0.50	0.00	12.64	0.00
Ethiopia	0.57	0.00	99.43	0.00	0.00	0.00	0.00	0.00	0.00	0.00	0.00	0.00
Central Africa	35.46	0.91	11.69	6.70	0.00	6.11	11.69	3.26	1.27	1.59	21.33	0.00
Southern Africa	0.64	0.38	98.47	0.12	0.00	0.04	0.10	0.00	0.01	0.00	0.23	0.00
Global	25.44	11.97	49.06	2.57	0.00	0.68	4.41	0.11	0.09	0.05	5.62	0.01

D. *pol* reassignment

Region	Proportion (%) of HIV-1 infections attributed to HIV-1 variants											
	A	B	C	D	E	F	G	H	J	K	URFs	Un-reassigned CRFs
Caribbean	0.36	94.41	0.40	1.28	0.00	0.01	1.71	0.07	0.01	0.00	1.64	0.11
Latin America	0.10	77.33	7.62	0.06	0.00	4.94	0.14	0.02	0.00	0.00	9.81	0.00
Western and Central Europe and North America	3.24	82.37	4.19	0.38	0.00	2.20	5.36	0.03	0.05	0.04	1.98	0.16
Eastern Europe & Central Asia	74.27	16.83	5.80	0.01	0.00	0.00	1.73	0.00	0.00	0.00	1.36	0.00
South Asia	0.96	1.18	94.76	0.00	0.00	0.00	0.00	0.00	0.00	0.00	3.11	0.00
South-East Asia	74.16	17.72	1.94	0.24	0.00	0.00	0.39	0.00	0.00	0.00	5.55	0.00
East Asia	46.73	19.57	28.60	0.01	0.00	0.01	0.17	0.00	0.00	0.00	4.92	0.00
Oceania	4.42	32.72	61.21	0.10	0.00	0.18	0.77	0.00	0.00	0.00	0.60	0.00
Middle East & North Africa	37.25	20.17	11.09	17.18	0.00	0.32	10.25	0.00	0.00	0.00	3.74	0.00
West Africa	3.77	0.02	1.05	0.75	0.00	0.56	78.69	0.05	0.86	0.00	14.26	0.00
East Africa	54.34	0.25	14.34	17.13	0.00	0.08	0.73	0.00	0.50	0.00	12.64	0.00
Ethiopia	0.57	0.00	99.43	0.00	0.00	0.00	0.00	0.00	0.00	0.00	0.00	0.00
Central Africa	19.29	1.11	11.69	6.70	0.00	6.11	24.76	3.26	4.16	1.59	21.33	0.00
Southern Africa	0.52	0.38	98.47	0.12	0.00	0.04	0.22	0.00	0.01	0.00	0.23	0.00
Global	16.88	12.03	49.07	2.55	0.00	0.66	12.74	0.11	0.30	0.05	5.62	0.01

E. *env* reassignment

Region	Proportion (%) of HIV-1 infections attributed to HIV-1 variants											
Region	A	B	C	D	E	F	G	H	J	K	URFs	Un-reassigned CRFs
Caribbean	1.50	94.41	0.40	0.18	0.02	0.03	1.18	0.53	0.01	0.00	1.64	0.11
Latin America	0.19	77.25	7.62	0.02	0.00	5.07	0.03	0.02	0.00	0.00	9.81	0.00
Western and Central Europe and North America	5.56	82.74	4.19	0.35	0.97	2.21	1.70	0.06	0.04	0.03	1.98	0.16
Eastern Europe & Central Asia	76.00	16.83	5.80	0.01	0.00	0.00	0.01	0.00	0.00	0.00	1.36	0.00
South Asia	0.96	1.18	94.76	0.00	0.00	0.00	0.00	0.00	0.00	0.00	3.11	0.00
South-East Asia	6.55	17.83	1.94	0.24	67.75	0.00	0.14	0.00	0.00	0.00	5.55	0.00
East Asia	0.16	19.42	28.60	0.01	46.83	0.01	0.04	0.01	0.00	0.00	4.92	0.00
Oceania	1.19	32.83	61.21	0.10	3.68	0.18	0.22	0.00	0.00	0.00	0.60	0.00
Middle East & North Africa	40.91	20.29	11.09	17.18	0.66	0.19	5.94	0.00	0.00	0.00	3.74	0.00
West Africa	51.65	0.07	1.05	0.66	0.09	0.63	30.55	0.18	0.86	0.00	14.26	0.00
East Africa	53.80	0.25	14.34	17.22	0.61	0.08	0.56	0.00	0.50	0.00	12.64	0.00
Ethiopia	0.57	0.00	99.43	0.00	0.00	0.00	0.00	0.00	0.00	0.00	0.00	0.00
Central Africa	30.92	1.16	11.69	6.37	1.16	6.19	9.90	5.52	4.16	1.59	21.33	0.00
Southern Africa	0.63	0.38	98.47	0.12	0.00	0.04	0.10	0.00	0.01	0.00	0.23	0.00
Global	19.24	12.06	49.06	2.53	5.31	0.68	4.95	0.20	0.30	0.05	5.62	0.01

Supplementary Table 3. Global and regional HIV-1 subtype distributions before and after reassignment of CRFs to 'pure' HIV-1 subtypes. Proportions of HIV-1 subtypes in each region and globally, based on the most recent available data for each country, before reassignment (A) or after reassignment of CRFs to 'pure' HIV-1 subtypes based on full-length sequence (B) or genome regions *gag* (C), *pol* (D), and *env* (E). Un-reassigned CRFs are CRFs for which reassignment was not possible as no relevant data was available (see Supplementary Table 1). This data underlies Figures 1 and 2 and Supplementary Figure 2.

Supplementary Table 4.

		Subtype-specific doses per region or globally (millions)										
Type of vaccine	Region	A	B	C	D	E	F	G	H	J	K	Total
Therapeutic	Caribbean	0.00	0.29	0.00	0.00	0.00	0.00	0.00	0.00	0.00	0.00	0.29
	Latin America	0.00	1.73	0.00	0.00	0.00	0.00	0.00	0.00	0.00	0.00	1.73
	Western and Central Europe and North America	0.02	2.04	0.00	0.00	0.00	0.02	0.04	0.00	0.00	0.00	2.12
	Eastern Europe & Central Asia	1.51	0.00	0.00	0.00	0.00	0.00	0.00	0.00	0.00	0.00	1.51
	South Asia	0.00	0.00	1.74	0.00	0.00	0.00	0.00	0.00	0.00	0.00	1.74
	South-East Asia	1.86	0.06	0.01	0.00	0.00	0.00	0.00	0.00	0.00	0.00	1.92
	East Asia	0.88	0.04	0.00	0.00	0.00	0.00	0.00	0.00	0.00	0.00	0.92
	Oceania	0.00	0.03	0.05	0.00	0.00	0.00	0.00	0.00	0.00	0.00	0.07
	Middle East & North Africa	0.07	0.03	0.00	0.06	0.00	0.00	0.01	0.00	0.00	0.00	0.16
	West Africa	4.44	0.00	0.00	0.00	0.00	0.00	0.00	0.00	0.00	0.00	4.44
	East Africa	4.82	0.00	0.09	0.00	0.00	0.00	0.00	0.00	0.00	0.00	4.91
	Ethiopia	0.00	0.00	0.71	0.00	0.00	0.00	0.00	0.00	0.00	0.00	0.71
	Central Africa	0.66	0.00	0.28	0.00	0.00	0.00	0.00	0.00	0.00	0.00	0.94
	Southern Africa	0.00	0.00	13.61	0.00	0.00	0.00	0.00	0.00	0.00	0.00	13.61
	Global	14.25	4.22	16.50	0.06	0.00	0.02	0.06	0.00	0.00	0.00	35.10
Prophylactic	Caribbean	0.00	22.17	0.00	0.00	0.00	0.00	0.00	0.00	0.00	0.00	22.17
	Latin America	0.00	364.37	0.00	0.00	0.00	1.50	0.00	0.00	0.00	0.00	365.87
	Western and Central Europe and North America	38.99	419.36	0.00	0.00	0.00	1.48	40.41	0.00	0.00	0.00	500.24
	Eastern Europe & Central Asia	144.33	4.24	0.00	0.00	0.00	0.00	0.00	0.00	0.00	0.00	148.57
	South Asia	0.00	0.00	828.76	0.00	0.00	0.00	0.00	0.00	0.00	0.00	828.76
	South-East Asia	447.18	63.81	106.52	0.00	0.00	0.00	0.00	0.00	0.00	0.00	617.52
	East Asia	850.66	90.03	0.00	0.00	0.00	0.00	0.00	0.00	0.00	0.00	940.69
	Oceania	0.00	15.32	5.48	0.00	0.00	0.00	0.00	0.00	0.00	0.00	20.80
	Middle East & North Africa	56.34	31.20	0.00	23.24	0.00	0.00	24.56	0.00	0.00	0.00	135.33
	West Africa	173.71	0.00	0.00	0.00	0.00	0.00	0.35	0.00	0.00	0.00	174.06
	East Africa	111.05	0.00	6.26	0.00	0.00	0.00	0.00	0.00	0.00	0.00	117.31
	Ethiopia	0.00	0.00	61.29	0.00	0.00	0.00	0.00	0.00	0.00	0.00	61.29
	Central Africa	76.20	0.00	21.52	0.00	0.00	0.00	0.00	0.00	0.00	0.00	97.72
	Southern Africa	0.00	0.00	87.05	0.00	0.00	0.00	0.00	0.00	0.00	0.00	87.05
	Global	1898.45	1010.52	1116.87	23.24	0.00	2.97	65.31	0.00	0.00	0.00	4,117.36

Supplementary Table 4. Regional and global estimates of number of doses of subtype-specific therapeutic and prophylactic HIV-1 vaccines. Estimates are based on the 'most common subtype' scenario and CRFs reassigned based on the full-length genome. This data underlies Figure 4.

WHO-UNAIDS Network for HIV Isolation and Characterisation

Alash'le G Abimiku, Simon Agwale, Chris Archibald, Boaz Avidor, María Gabriela Barbás, Francoise Barre-Sinoussi, Banson Barugahare, El Hadj Belabbes, Silvia Bertagnolio, Deborah Birx, Aleksei F Bobkov, James Brandful, Helba Bredell, Catherine A Brennan, James Brooks, Marie Bruckova, Luigi Buonaguro, Franco Buonaguro, Stefano Buttò, Anne Buvé, Mary Campbell, Jean Carr, Alex Carrera, Manuel Gómez Carrillo, Connie Celum, Beth Chaplin, Macarthur Charles, Dimitrios Chatzidimitriou, Zhiwei Chen, Katsumi Chijiwa, David Cooper, Philip Cunningham, Anoumou Dagnra, Cillian F de Gascun, Julia Del Amo, Elena Delgado, Ursula Dietrich, Dominic Dwyer, Dennis Ellenberger, Barbara Ensoli, Max Essex, Hervé Fleury, Peter N Fonjungo, Vincent Foulongne, Deepak A Gadkari, Feng Gao, Federico García, Roger Garsia, Guy Michel Gershy-Damet, Judith R Glynn, Ruth Goodall, Zehava Grossman, Monick Lindenmeyer Guimarães, Beatrice Hahn, Raph L Hamers, Osamah Hamouda, Ray Handema, Xiang He, Joshua Herbeck, David D Ho, Africa Holguin, Mina Hosseinipour, Gillian Hunt, Masahiko Ito, Mohamed Ali Bel Hadj Kacem, Erin Kahle, Pontiano Kaleebu, Marcia Kalish, Adeeba Kamarulzaman, Chun Kang, Phyllis Kanki, Edward Karamov, Jean-Claude Karasi, Kayitesi Kayitenkore, Tony Kelleher, Dwip Kitayaporn, Leondios G Kostrikis, Claudia Kucherer, Claudia Lara, Thomas Leitner, Kirsi Liitsola, Jai Lingappa, Marek Linka, Ivette Lorenzana de Rivera, Vladimir Lukashov, Shlomo Maayan, Luzia Mayr, Francine McCutchan, Nicolas Meda, Elisabeth Menu, Fred Mhalu, Doreen Mloka, John L Mokili, Brigitte Montes, Orna Mor, Mariza Morgado, Fausta Mosha, Awatef Moussi, James Mullins, Rafael Najera, Mejda Nasr, Nicaise Ndembi, Joel R Neilson, Vivek R Nerurkar, Florian Neuhann, Claudine Nolte, Vlad Novitsky, Philippe Nyambi, Marianna Ofner, Fem J Paladin, Anna Papa, Jean Pape, Neil Parkin, Chris Parry, Martine Peeters, Alexandra Pelletier, Lucía Pérez-Álvarez, Deenan Pillay, Angie Pinto, Trinh Duy Quang, Cecilia Rademeyer, Filimone Raikanikoda, Mark A. Rayfield, Jean-Marc Reynes, Tobias Rinke de Wit, Kenneth E Robbins, Morgane Rolland, Christine Rousseau, Jesus Salazar-Gonzales, Hanan Salem, Mika Salminen, Horacio Salomon, Paul Sandstrom, Mario L Santiago, Abdoulaye D Sarr, Bryan Schroeder, Michel Segondy, Philippe Selhorst, Sylvester Sempala, Jean Servais, Ansari Shaik, Yiming Shao, Amine Slim, Marcelo A Soares, Elijah Songok, Debbie Stewart, Julie Stokes, Shambavi Subbarao, Ruengpung Sutthent, Jun Takehisa, Amilcar Tanuri, Kok Keng Tee, Kiran Thapa, Michael Thomson, Tyna Tran, Willy Urassa, Hiroshi Ushijima, Philippe van de Perre, Guido van der Groen, Kristel van Laethem, Joep van Oosterhout, Ard van Sighem, Eric van Wijngaerden, Anne-Mieke Vandamme, Jurgen Vercauteren, Nicole Vidal, Lesley Wallace, Carolyn Williamson, Dawit Wolday, Jianqing Xu, Chunfu Yang, Linqi Zhang, Rong Zhang.

Affiliations

Institute of Human Virology, University of Maryland, Baltimore, MD, USA (A G Abimiku, J Carr); Gede Foundation, Abuja, Nigeria (S Agwale); Public Health Agency of Canada, Ottawa, ON, Canada (C Archibald, J Brooks, M Ofner, P Sandstrom, J Stokes); Tel-Aviv Sourasky Medical Center, Tel-Aviv, Israel (B Avidor); Ministerio de Salud, Córdoba, Argentina (M G Barbás); Institut Pasteur, Paris, France (F Barre-Sinoussi, E Menu); Ministry of Health, Entebbe, Uganda (B Barugahare); National Reference Laboratory on HIV/AIDS, Institut Pasteur d'Algérie, Algiers, Algeria (E Belabbes); World Health Organization, Geneva, Switzerland (S Bertagnolio); Office of the Global AIDS Coordinator, Washington, DC, USA (D Birx); The D I Ivanovsky Institute of Virology, Moscow, Russia(A F Bobkov); Noguchi Memorial Institute for Medical Research, University of Ghana, Accra, Ghana (J Brandful); University of Cape Town, Cape Town, South Africa (H Bredell, C Rademeyer, P Selhorst, D Stewart, C Williamson); Abbott Laboratories, Chicago, IL, USA (C A Brennan); National Institute of Public Health, Prague, Czech Republic (M Bruckova, M Linka); AIDS Reference Center, National Cancer Institute "Fond. G. Pascale", Naples, Italy (L Buonaguro, F Buonaguro); National AIDS Center, Istituto Superiore di Sanità, Rome, Italy (S Buttò, B Ensoli); Institute of Tropical Medicine, Antwerp, Belgium (A Buvé, G van der Groen); University of Washington School of Medicine, Seattle, WA, USA (M Campbell, C Celum, J Herbeck, E Kahle, J Lingappa, J Mullins, M Rolland, C Rousseau); St Vincent's Hospital, Sydney, Australia (A Carrera, P Cunningham); University of Buenos Aires, Buenos Aires, Argentina (M Carrillo, H Salomon); Harvard T H Chan School of Public Health, Boston, MA, USA (B Chaplin, P Kanki); Gheskio Center, Port-au-Prince, Haiti (M Charles, C Nolte, J Pape); Aristotle University of Thessaloniki, Thessaloniki, Greece (D Chatzidimitriou, A Papa); Chinese Academy of Medical Sciences, Peking Union Medical School, Beijing, China (Z Chen, L Zhang, R Zhang); Fukuoka Institute of Health and Environmental Sciences, Kyushu University Hospital, Dazaifu, Japan (K Chijiwa); The Kirby Institute, Sydney, NSW, Australia (D Cooper, T Kelleher, A Pinto, A Shaik); Faculté des Sciences de la Santé, Université de Lomé, Lomé, Togo (A Dagnra); University College Dublin, Dublin, Ireland (C de Gascun); Instituto de Salud Carlos III, Madrid, Spain (J Del Amo, E Delgado, R Najera, L Pérez-Álvarez, M Thomson); Chemotherapeutisches Forschungsinstitut, Georg-Speyer-Haus, Frankfurt, Germany (U Dietrich); Pathology West, Westmead Hospital, Westmead, NSW, Australia (D Dwyer, K Thapa, T Tran); Centers for Disease Control and Prevention, Atlanta, GA, USA (D Ellenberger, P N Fonjungo, M A Rayfield, K E Robbins, S Subbarao, C Yang); Harvard School of Public Health, Boston, MA, USA (M Essex, V Novitsky, A D Sarr); Duke University

Medical Center, Durham, NC, USA (F Gao); University of Bordeaux, Bordeaux, France (H Fleury); Montpellier University Hospital, Montpellier, France (V Foulongne, P van de Perre); National AIDS Research Institute, Pune, India (D A Gadkari); Complejo Hospitalario Universitario de Granada, Granada, Spain (F García); Royal Prince Alfred Hospital, Sydney, Australia (R Garsia, H Salem); HIV Laboratory Programme on AIDS/AFRO, World Health Organisation, Ouagadougou, Burkina Faso (G M Gershy-Damet); London School of Hygiene and Tropical Medicine, London, UK (J R Glynn); University College London, London, UK (R Goodall); National HIV Reference Laboratory, Ministry of Health, Tel Aviv, Israel (Z Grossman, O Mor); Instituto Oswaldo Cruz, FIOCRUZ, Rio de Janeiro, Brazil (M L Guimarães, M Morgado); University of Pennsylvania, Philadelphia, PN, USA (B Hahn); Amsterdam Institute for Global Health and Development, Amsterdam, Netherlands (R L Hamers, T Rinke de Wit); Robert Koch Institute, Berlin, Germany (O Hamouda, C Kucherer); Yamanashi Medical University, Yamanashi, Japan (R Handema, M Ito); National Center for AIDS/STD Control and Prevention, China CDC, Beijing, China (X He, Y Shao, J Xu); Aaron Diamond AIDS Research Center, The Rockefeller University, New York, NY, USA (D D Ho, L G Kostrikis); Ramón y Cajal Research Institute, Hospital Universitario Ramón y Cajal de Madrid, Madrid, Spain (A Holguin); University of North Carolina, Chapel Hill, NC, USA (M Hosseinipour); National Institute for Communicable Diseases, Johannesburg, South Africa (G Hunt); Charles Nicolle Hospital, Tunis, Tunisia (M Kacem, A Moussi, M Nasr, A Slim); Medical Research Council, Entebbe, Uganda (P Kaleebu, C Parry); Vanderbilt Institute for Global Health, Vanderbilt University School of Medicine, Nashville, TN, USA (M Kalish); University of Malaya, Kuala Lumpur, Malaysia (A Kamarulzaman, K-K Tee); Institute for Molecular Biology and Genetics and Medical College, Seoul National University, Seoul, Korea (C Kang); Gamaleya Center for Epidemiology and Microbiology, Moscow, Russian (E Karamov); National Reference Laboratory, Kigali, Rwanda (J-C Karasi); Emory University School of Medicine, Atlanta, GA, USA (K Kayitenkore); HIV/AIDS Collaboration, Nonthaburi, Thailand (D Kitayaporn); Karolinska Institute, Huddinge University Hospital, Stockholm, Sweden (C Lara); Los Alamos National Laboratory, Los Alamos, NM, USA (T Leitner); National Institute for Health and Wellfare, Helsinki, Finland (K Liitsola, M Salminen); National Autonomous University of Honduras, Tegucigalpa, Honduras (I Lorenzana de Rivera); Academic Medical Center, University of Amsterdam, Amsterdam, Netherlands (V Lukashov); Hadassah U Hospital, Jerusalem, Israel (S Maayan); New York University School of Medicine, New York, NY, USA (L Mayr, P Nyambi); Henry M Jackson Foundation for the Advancement of Military Medicine, Bethesda, MD, USA (F McCutchan);

Centre Muraz, Bobo-Dioulasso, Burkina Faso (N Meda); Muhimbili University of Health Sciences, Dar-es-salaam, Tanzania (F Mhalu, D Mloka, F Mosha, W Urassa); University of Edinburgh, Edinburgh, UK (J L Mokili); Montpellier University Hospital, Montpellier, France (B Montes, M Segondy); Institute of Human Virology, Abuja, Nigeria (N Ndembi); University of Washington, Seattle, WA, USA (J R Neilson); University of Hawaii, Honolulu, HI, USA (V R Nerurkar); University Clinic Heidelberg, Heidelberg, Germany & Lighthouse Trust, Lilongwe, Malawi (F Neuhann); Research Institute for Tropical Medicine, Muntinlupa City, Manila, Philippines (F J Paladin, M L Santiago); Data First Consulting, Inc, Belmont, CA, USA (N Parkin); University of Montpellier, Montpellier, France (M Peeters, N Vidal); Centre de Recherche Public-Santé, Luxembourg, Luxembourg (A Pelletier, J Servais); Africa Health Research Institute, Durban, KwaZulu-Natal, South Africa & Division of Infection and Immunity, University College London, London, UK (D Pillay); Institute of International Health, University of Tokyo, Tokyo, Japan (T D Quang); University of Sydney, Sydney, NSW, Australia (F Raikanikoda); Institut Pasteur du Cambodge, Phnom Penh, Cambodia (J-M Reynes); University of Alabama at Birmingham, Birmingham, AL, USA (J Salazar-Gonzales); Auckland City Hospital, Auckland, New Zealand (B Schroeder); Uganda Virus Research Institute, Entebbe, Uganda (S Sempala); Instituto Nacional de Câncer, Rio de Janeiro, Brazil (M A Soares); Kenya Medical Research Institute, Nairobi, Kenya (E Songok); National HIV Repository and Bioinformatic Center, Siriraj Hospital, Mahidol University, Thailand (R Sutthent); Laboratory of Viral Pathogenesis, Kyoto University, Kyoto, Japan (J Takehisa); Federal University of Rio de Janeiro, Rio de Janeiro, Brazil (A Tanuri); Aino Health Science Center and Aino University, Tokyo, Japan (H Ushijima); Rega Institute for Medical Research, KU Leuven, Belgium (K van Laethem, E van Wijngaerden, A-M Vandamme, J Vercauteren); Department of Medicine, Blantyre, Malawi (J van Oosterhout); Stichting HIV Monitoring, Amsterdam, Netherlands (A van Sighem); Health Protection Scotland, Glasgow, UK (L Wallace); and Ethiopian Health & Nutrition Research Institute, Addis Ababa, Ethiopia (D Wolday).

References

1. Paraskevis D, Magiorkinis M, Vandamme A-M, Kostrikis LG, Hatzakis A. Re-analysis of human immunodeficiency virus type 1 isolates from Cyprus and Greece, initially designated 'subtype I', reveals a unique complex A/G/H/K/? mosaic pattern. Journal of General Virology. 2001;82(3):575-80.
2. McCutchan FE. Understanding the genetic diversity of HIV-1. AIDS. 2000;14(3):S31-S44.
3. Visawapoka U, Tovanabutra S, Currier JR, Cox JH, Mason CJ, Wasunna M, et al. Circulating and unique recombinant forms of HIV type 1 containing subsubtype A2. AIDS Research & Human Retroviruses. 2006;22(7):695-702.
4. Yamaguchi J, Badreddine S, Swanson P, Bodelle P, Devare SG, Brennan CA. Identification of new CRF43_02G and CRF25_cpx in Saudi Arabia based on full genome sequence analysis of six HIV type 1 isolates. AIDS research and human retroviruses. 2008;24(10):1327-35.
5. Fernandez-Garcia A, Perez-Alvarez L, Cuevas MT, Delgado E, Munoz-Nieto M, Cilla G, et al. Identification of a new HIV type 1 circulating BF intersubtype recombinant form (CRF47_BF) in Spain. AIDS research and human retroviruses. 2010;26(7):827-32.
6. Foster GM, Ambrose JC, Hue S, Delpech VC, Fearnhill E, Abecasis AB, et al. Novel HIV-1 recombinants spreading across multiple risk groups in the United Kingdom: the identification and phylogeography of Circulating Recombinant Form (CRF) 50_A1D. PLoS One. 2014;9(1): e83337.
